# Control of Unfair Terms Under Cypriot Contract Law

**DOI:** 10.1007/s10991-022-09304-8

**Published:** 2022-07-14

**Authors:** Nicholas Mouttotos

**Affiliations:** Institute for Commercial Law, Faculty of Law, University of Bremen, Bremen, Germany

**Keywords:** Contract Law, Mixed jurisdictions, Good faith, Consumer Law, Common Law

## Abstract

The introduction of the EU Unfair Terms in Consumer Contracts Directive in the legal system of Cyprus was the first instrument specifically dealing with the problems that arise with standard form contracts and the issue of consent. However, the Directive only generated caselaw approximately twenty years after its adoption, whereas the principle-based approach of the test for unfairness (significant imbalance test and good faith requirement) was interpreted in the common law notion of absence of dishonesty. Hence, Cypriot courts interpreted good faith as “honesty in fact”. Under this interpretation, it seems that both procedural and substantive unfairness are not captured under the test. It is understood that whereas the statute codifying the contract law of Cyprus has incorporated the [civil law] will theory of contracts, no tools for the invalidation and scrutiny of terms that may be deemed unfair were provided. The statute placed considerable emphasis on procedural fairness, however, Cypriot courts seemed unwilling to adapt their approach and deal with the problems that standard form contracting poses, failing also to follow the developments in the UK. Thus, even after the adoption of EU consumer law directives, Cypriot courts show an unwillingness to scrutinize the parties’ initial allocation of risk, even though such allocation was done in a standardized manner by one party. Despite the mixed character of the legal system, consumer law directives and the introduction of content control can be seen as legal irritants.

## Introduction

The most impactful EU legislative measure upon the common law of contract is the Directive on Unfair Terms in Consumer Contracts (Directive 93/13)^1^ which has posed the most challenges. This is arguably due to the orientation of English contract law towards adopting a more freedom of contract approach (MacMillan 2016; Mouttotos 2019). However, while in the UK there have been cases where a duty of good faith was recognized as an implied term within the category of ‘relational contracts’,^2^ and some English authorities have also imposed a special notice requirement, reminiscent of the principle of good faith,^3^ it is still not possible to consider this as a shift towards the continental use of the concept, which is now less likely to take place given the UK’s exit from the EU.^4^

Contract law in Cyprus is based on the common law principle that contracts are commonly formed by offer and acceptance. A principle underpinning contract law in Cyprus provides that where written agreements exist, those agreements are the basis to deduce and derive the intentions of the parties.^5^ The tension with the good faith requirement and the control for unfairness is, thus, apparent in the case of Cyprus, where the approach of the courts is stricter than the one prevalent in English law.^6^ Cypriot courts’ have given the good faith requirement a common law gloss by focusing on an absence of dishonesty in agreeing upon the terms. Moreover, their approach towards issues of contract formation, such as whether contractual consent was given upon specific terms, is based on the rule in *L’Estrange v Graucob*, where it was held that where a document containing contractual terms was signed, then, in the absence of fraud, misrepresentation and *non est factum*, the party cannot claim that he or she has not read the document and is, therefore, bound by such terms.^7^ This is despite certain provisions of the Contracts’ Law (Cap. 149) being influenced by the ‘will theory’ of contracts and the civilian understanding of consent.^8^ This paper will evaluate the introduction of content control of terms in Cyprus by an analysis of the caselaw of Cypriot courts. A large number of claims from debtors for mis-selling practices by financial institutions have come before courts in the last decade, while the lockdown measures introduced to slow the spread of the COVID-19 pandemic have created further problems in the performance of contracts.^9^

This paper firstly introduces the legal system of Cyprus, presenting its unique nature as a mixed legal system in which common law and civil law contend for supremacy. Secondly, the paper proceeds with a brief analysis of contract law in Cyprus which is codified in the Contracts’ Law (Cap. 149), a transplant of the Indian Contract Act of 1872. The importance of the English common law in the development of the law of contract in Cyprus is mentioned, emphasizing the unwillingness, however, to follow the discussions in England and Wales as to the role of a duty of good faith in the common law of contract. A comparative analysis of the way content control of terms has been performed in the two jurisdictions, namely, England (and Wales) and Cyprus is performed. It will be seen that a functional equivalent of the content control of standard form contracts found in the English common law is not to be found under Cyprus contract law. The paper presents the tension between European law, particularly Directive 93/13, and the common law of contract before examining the interpretation that the Cypriot courts have given to the requirement of good faith found within the directive and reaching conclusions as to the role of good faith (and interpretation of it) within the common law of contract regime in Cyprus.

## The Legal System of Cyprus and the Influence of the English Common Law

In order to have a better understanding of the greater context it is worth mentioning that the legal system of Cyprus is a mixed legal system that, as Symeon Symeonides correctly points out, owes the origin and survival of its “diverse elements […] to its troubled political history” (Symeonides 2003, p.454). These elements “are accidents of history” (Symeonides 2003, p. 454). Cyprus’ long periods of foreign occupation, which is still present on the island, have formed and are forming its legal system. Firstly, by the inheritance of the common law system and, secondly, by the prevailing perception that the legal system is in an interim stage until a political settlement is reached and the Turkish Cypriots return in the bi-communal form of government. Therefore, the malfunctions of the ‘Cyprus problem’^10^ have spilled over in the legal system, while it is uncertain what the impact of a future political settlement will be. These problems might present an opportunity, as they make the legal system of Cyprus intellectually intriguing for comparative law scholars. The journey of mixedness of Cyprus law has taken a “contrariwise movement”, since the common law has emigrated rather than migrated as in the majority of mixed jurisdictions (Palmer 2012, p. 9).

Compared to other mixed jurisdictions, the dual foundations upon which such mixed systems are built upon, namely common law and civil law materials, are reversely allocated in the case of Cyprus (Palmer 2012; Parise 2012; Mouttotos 2019). This adds to the theory of mixed jurisdiction a ‘juridical unicorn’, i.e. common law is ‘cordoned off’ in the field of private law with the exception of non-commercial matters and criminal law, whereas public law is based on civil law materials (Palmer 2012, p. 9). Procedural law follows common law as in most other mixed jurisdictions (Goldstein 2004); nevertheless, in the case of Cyprus thinking in common law terms is vital in order to better understand the law (Hatzimihail 2017; Hatzimihail, 2013, p. 40; Mouttotos 2019). The common law and equity are sources of law based on the Courts of Justice Law, unless otherwise provided for by law or the Constitution. Thus, this provision confirmed the colonial *status quo*.^11^ Hatzimihail highlights that the empowerment of a people attached to motherlands and languages which fall firmly within the continental legal tradition could have led Cyprus away from the common law tradition (Hatzimihail, 2015; Hatzimihail, 2013, p. 45; Symeonides 2003). However, as former Judge Pikis points out:

“The inheritance of English law in Cyprus had positive effects in relation to human rights, including property rights. It is no coincidence that despite the blows inflicted upon Cyprus in 1974, the rule of law retained its force, the State survived, helping in the sustenance of the Republic of Cyprus becoming in due course a member of the European Union” (Pikis, 2017).

Cyprus, today, more closely resembles a common law jurisdiction than other mixed jurisdictions, even though the civil law influence is constantly expanding in new areas of the law (Mouttotos 2019). What is more, most of the legislation, especially in private law at large, has been imported or transplanted from abroad. According to Hatzimihail, this importation has often resulted in veritable transplants in some areas of the law, while in other instances resulted in the formation of altered legal regimes (Hatzimihail 2017), evoking Gunther Teubner’s idea of legal irritants (Teubner 1998).

An example, relevant for the purposes of this article, is the Unfair Contract Terms Directive (Directive 93/13)^12^ where the courts have given the good faith requirement provided under Article 3 of the directive a common law gloss by focusing on an absence of dishonesty in agreeing upon the terms. Good faith by the seller or supplier is seen as a *praesumptio iuris tantum*.^13^ The burden is upon the consumer to disprove the existence of good faith.^14^ This can arguably be attributed to the lack of understanding of the principle-based approach of Directive 93/13 that requires courts to consider both core requirements in assessing the fairness of contractual terms, i.e. the significant imbalance test and the requirement of good faith. In particular, Article 3 of the directive provides that “[a] contractual term which has not been individually negotiated shall be regarded as unfair if, contrary to the requirement of good faith, it causes a significant imbalance in the parties’ rights and obligations arising under the contract, to the detriment of the consumer”.^15^ Thus, there are two requirements for a finding of a term as unfair along with the detriment suffered by the consumer: the existence of a significant imbalance between the parties, and it being contrary to good faith (Howells 2017, p. 1916). Cyprus courts took the second requirement of the Directive in isolation thus examining evidence showing bad faith or dishonesty in the conclusion of the contract.

Unlike other common law jurisdictions where more settled approaches to good faith can be seen (Hogg, 2017), Cyprus does not recognize an overriding principle of good faith in the formation and performance of contracts.^16^ The law imposes a negative duty on negotiating parties to refrain from making misrepresentations.^17^ Positive disclosure and fiduciary duties are only required where parties are in a confidential relationship, and the person upon whom confidence is reposed ought to make full disclosure of all material facts in respect of any contract he or she will make with the other party. As a result of the good faith requirement imposed under EU consumer law, the courts developed an underlying principle of subjective good faith, interpreting the requirement as an absence of dishonesty. In other words, good faith is seen as honesty in fact (Farnsworth 1963).

The impact of EU derivative law is just beginning to be felt in contractual practice and caselaw, mainly through Directive 93/13. Despite its early adoption, prior to accession, the Directive has only recently started to exert influence. Courts altered their approach in a minimal number of cases and scrutinized terms in consumer contracts. As Hatzimihail highlights, effective consumer protection is still in its early stages (Hatzimihail 2017). This contrasts with the inroads that the principle of good faith has made into English caselaw.^18^ The reasoning in most contract cases in Cyprus, however, is not comparable to English appellate court opinions (Hatzimihail 2017). In any case, it is conceivable that Cyprus courts may deviate from English common law since the Supreme Court of Cyprus is the court of last resort in all legal questions, apart from international and European law, whereas the persistence of British influence on Cyprus and respect for English law does not necessarily stem from an emotional or metropolitan bond (Hatzimihail, 2015). It can be argued that such a deviation is hindered by the lack of a superior appellate court of last resort that can decide only on cases of importance (Hatzimihail, 2015). This is what impedes the development of a consistent Cyprus caselaw distinct from the English paradigm (Hatzimihail, 2015). An intermediate appellate jurisdiction (which is to be introduced in the near future) will allow Supreme Court judges more time for reasoning in depth and increase the quality of judicial reasoning.

### Cap. 149 and the “Will Theory” of Contracts

This section will address the theoretical framework upon which Cap. 149 was drafted and mention the tools that the Contracts’ Law provided for controlling unfair terms, even though in practice the scrutiny of terms was of a limited extent. Even though the Contracts’ Law was influenced by the will theory of contracts, emphasis was placed on the outward behavior of assent through the signature of the document.^19^ In general, Cyprus contract law is patterned after English statutory models with new legislation in contract law simply replacing previous statutory transplants. (Hatzimihail 2017).^20^ Moreover, legislative deviations from the English paradigm are only absorbed very gradually in legal practice and in caselaw (Hatzimihail 2017; Mouttotos 2019).^21^. This is evidenced by the ratification of the United Nations Convention on Contracts of the International Sale of Goods (CISG),^22^ which is yet to generate any caselaw, as well as statutes implementing EU derivative law, such as Directive 93/13, despite the fact that it was transposed into law prior to accession to the EU(Hatzimihail 2017; Mouttotos 2019).^23^ The law of contract in Cyprus is contained in Chap. 149 of the Laws of Cyprus originally enacted in 1957 (also known as Cap. 149) being effectively a transplant of the Indian Contract Act of 1872.^24^ Under Sect. 2 of Cap. 149 a general rule of construction is established, whereby the Contracts’ Law shall be interpreted in accordance with the principles of legal interpretation obtained from England, and expressions used in it shall be presumed to be used with the meaning attached to them in English law, so far as is consistent with their context, and except as may be otherwise expressly provided.^25^

Tofaris indicates that the Indian Contract Act formulated a single law of contract combining common law and equity doctrines within a common framework (Tofaris, 2020). As a product of its time, the Indian Contract Act emulated the classical model of contract law that was prevalent in England (Tofaris, 2020). However, it moved beyond that in enshrining the ‘will theory’ of contracts (Tofaris, 2020; Ibbetson 2001; Kennedy 2000). Section 10 of the Act is seen as an explicit statement of the will theory: “All agreements are contracts if they are made by the free consent of parties competent to contract, for lawful consideration and with a lawful object, and are not hereby expressly declared to be void” (Swain 2015, p. 265).^26^ This is a result of the influence civilian sources of the time had upon the drafting of the Indian Contract Act, which is reflected in the understanding of a contract as an agreement. The description of contract as an agreement is also recognized under the common law, however, in continental jurisdictions, it informs the way the subject is structured (Swain 2015, p. 265; Chloros 1959).

According to Cap. 149, two or more persons are said to consent when they agree upon the same thing in the same sense.^27^ This definition shows that Cap. 149 considered consent as a central concept of organization, and if consent was ‘free’ then the agreement was to be respected (Tofaris, 2020, p. 113). During the preparation of the Indian Contract Act, civilian sources played a major role through the writings of Robert Joseph Pothier (Swain 2015, p. 264-5; Tofaris, 2020; Pothier (Evans translation), 1806). Pothier’s views were the basis for the consensualist philosophy of the French law of contracts (Barnes 2008, p. 370; Chloros 1959). As Anne de Moor highlights, the analysis of the process of formation of contract in French law verifies the existence of a consensus between the parties, whereas under the English common law it verifies the existence of a promise in return for consideration (de Moor 1986, p. 276; Chloros 1968).

The fundamental question in contract law is whether the parties have mutually agreed to some exchange (Barnes 2008, p. 362). Mutual assent is analyzed objectively – through the external communications of assent as perceived by the other party – and subjectively – through the examination of the actual, literal intentions of the parties (Barnes 2008, p. 362). French contract law embraces the subjective theory as a result of the will theory of contracts, while the common law relies on the objective theory.^28^ The real intention of the parties is sacrosanct under French law; giving effect to such will is just, “since *ex hypothesi* the parties will not agree to anything harmful to themselves” (Chloros 1959, p. 615). Hence, contract scholars have stated that “[f]or a contract to exist in French law there must be a subjective agreement as to its terms[…]” (de Moor 1986, p. 279).^29^ The metaphysical effort to seek the *consensus ad idem* was, to a certain extent, limited by placing emphasis upon outward behavior (Chloros 1968, p. 147-8). Nonetheless, continental jurisdictions such as France have a more expansive conceptualization of the ‘will’ of the parties, than the one reflected with the statutory provision found in the Indian Contract Act.

Cap. 149 with Sect. 13 which establishes the concurrence of the wills, later on elaborated rules on coercion, undue influence, fraud, misrepresentation and mistake, ensuring that the parties’ consent is ‘free’ (Tofaris, 2020, p. 114).^30^ In that sense, emphasis was placed on procedural fairness with Cap. 149 essentially not providing the necessary tools for examining the harshness of the terms beyond these procedural safeguards. As Tofaris highlights, (with reference to the Indian Contract Act) the Act was less concerned about substantive fairness, putting more emphasis on procedural fairness through the elaborate rules on coercion, undue influence, fraud and misrepresentation (Tofaris, 2020, p. 114).^31^ However, unlike their Indian counterparts,^32^ the Cypriot courts were unwilling to control onerous terms through the tools provided under Cap. 149, thus dealing with the challenges that standard form contracting presented. The courts refused to delve deeper into the discussions about finding mutual assent in cases of standard form contracts, merely focusing on the external communication of intention through the signature on the document from the adhering party. Therefore, the implementation of the Directive 93/13 filled a lacuna by providing for the judicial control of unfair terms in consumer contracts, thus granting courts discretionary powers as to the interpretation and enforcement of the terms. The evident reluctance and uncomfortableness of Cypriot courts to use such discretionary powers is in part explained by the fact that Cyprus did not follow the enactment of the UK Unfair Contract Terms Act of 1977^33^ which imposed further limits on the extent to which, under the laws of England, Wales and Northern Ireland, civil liability for breach of contract, or for negligence or other breach of duty, can be avoided by means of contract terms and otherwise.^34^

### The Importance of the English Common Law for the Development of Contract Law in Cyprus

Article 29(1)(c) of the Courts of Justice Law provides that the applicable law in Cyprus is the common law and the principles of equity;^35^ however, codified legislation is the starting point for the development of judicially constructed rules, an element that underlines the hybrid nature of the law (Hatzimihail, 2015). The common law is thus regarded as binding, subject to contrary statutory provisions. The Contracts’ Law (Cap. 149) falls within the legislation enacted during the colonial era and maintained under Article 188(1) of the Constitution, in contrast to legislation enacted after independence (Hatzimihail, 2015).^36^ English common law rules that may be superseded by statute in England and Wales are still valid in Cyprus, such as, the doctrine of privity of contract and third-party rights (Hatzimihail, 2015).^37^

The primary differences between the Indian Contract Act and the Contracts’ Law (Cap. 149) are technical: certain explanations were moved into the main text, illustrations have been removed and specific performance is provided for in Cyprus contract law (Hatzimihail, 2015; Swaminathan 2018). The most important difference has to do with the interpretation of statute. In Cyprus a statute is to be interpreted in accordance with the principles of legal interpretation obtained in England and Wales. Also, expressions used in a statute are to be presumed, so far as is consistent with their context, to be used with the meaning attaching to them in English law.^38^ It should be pointed out, nonetheless, that certain provisions were read by Cyprus courts as deviating from the common law.^39^

Reference to English law by Cypriot courts is frequent, and Cypriot law is usually supplemented by English developments, however, with regard to the control of fairness of terms this has not been the case.^40^ In *Pastella Marine v Iranian Tanker*,^41^ the Supreme Court noted that in interpreting a statute it may resort to English law authorities interpreting analogous English statutes (Mouttotos 2019). In *Sekavin S.A. v Ship “PlatonCh”*,^42^ it was stipulated that Sect. 70 of the Cap. 149 reproduces common law in respect of quasi-contractual liability of recipient of goods or services supplied or rendered not gratuitously. According to the Court in *Saab and Another v Holy Monastery of Ayios Neophytos*,^43^ Section 73 of Cap. 149 aims to reproduce the common law rules on damages for breach of contract as they crystallized and were fashioned in the case of *Hadley v Baxendale*.^44^ Damages, therefore, aim to restore the party to the position he/she would be in, but for the breach.^45^

Although there are differences in the rules governing the making and performance of, or failure to perform, contracts in civil law and the common law of contract, the approach of Cypriot courts towards Directive 93/13 and the reception of certain rules contained therein as a legal irritant can also be explained by the differences in the analysis of formation of contract and the meaning that contractual consent takes in the common law of contract. As Anne de Moor highlighted, “[a]ccepting in English law means doing the act as requested or specified by the promisor with the intention of taking up the offer” (de Moor 1995, p. 262). In French law, for example, the analysis tries to verify the existence of an agreement or consensus between the parties in contradiction to the common law of contract where the analysis focuses on finding a promise in return for consideration (de Moor 1986). Thus, in the common law it is irrelevant whether the consumer has notice of the extent of the term; he or she need only know of the existence of the term before he/she can be said to have accepted it. In contrast, under civil law, consent requires an understanding of the substantive terms of the contract otherwise there can be no meeting of wills on them. Hence, the dual provisions of Article 5 and Article 4(2) of Directive 93/13 are closely oriented towards the civil law traditions since they require the transparency of terms (terms should be in plain and intelligible language), especially of substantive terms.^46^

English jurists are unwilling to conclude that there was a lack of acceptance, as a possible sanction for the disregard of the transparency requirement provided under Article 5 (de Moor 1995). This may explain the criticism by the Advocate General of the EU Court of Justice (CJEU), Gerard Hogan of the judgment of the CJEU in *Amazon.* 47 The latter decision elevated the transparency test found in Article 5 as an autonomous test for unfairness.^48^ Given the increased relevance of transparency in EU law in general, and Directive 93/13 in particular, when it comes to issues of assenting to the terms of the contract through the extension of Article 5, traditional contract law principles that apply in Cyprus are being challenged, such as the duty to read the terms of the contract. Despite a recent shift in the jurisprudence of the courts, most decisions examine the unfairness of terms by asking whether the seller or supplier exerted any pressure or undue influence on the consumer, with the latter having the burden of rebutting the presumption that the seller or supplier acted in good faith.^49^

### Standard form Contracts and the Control of Onerous Terms in the UK

It is vital to point out the different way standard form contracts were treated in the UK as compared to Cyprus, which can be attributed to the discretion placed upon courts as a result of the Unfair Contract Terms Act of 1977, but also resulting from developments in the caselaw. The 1977 Act conferred a substantial degree of discretion on a court in finding a particular exclusion or limitation clause to be unreasonable (McKendrick 2019). The Act enabled courts to control certain exclusion and limitation clauses unless the clause satisfied the requirement of reasonableness (Vogenauer 2013, p. 96). Section 11(1) defines “reasonableness” in broad terms: “the term shall have been a fair and reasonable one to be included having regard to the circumstances which were, or ought reasonably to have been, known to or in the contemplation of the parties when the contract was made”. Schedule 2 provides guidelines on how the reasonableness test is to apply. This involves the consideration of matters such as, *inter alia*, the strength of the bargaining positions of the parties; whether the customer was induced to agree to the term; and whether the customer knew or ought to reasonably have known of the existence of the term.^50^

In general, though, English courts have developed other common law techniques to control unfair contract terms and practices, such as restrictive rules on incorporation and interpretation of terms (Booysen, 2020, p. 362). Exemption clauses have also been interpreted restrictively by courts in order to limit their effects (Booysen, 2020, p. 363). Exemption clauses in Cyprus are also subject to the controls imposed by English courts in the judgment of *Gillespie Brothers & Co. Ltd*. *v Roy Bowles Transport Ltd. and Another*.^51^ In particular, the exemption clause should be clear and unambiguous and as a matter of construction it should be construed as not applying to a situation created by a fundamental breach of contract.^52^ In general, exemption clauses are respected if they are clearly worded, and in case of doubt as to their scope, are interpreted against the party invoking them. Lastly, exemption clauses do not cover negligent acts, unless this is unequivocally stated in their text.^53^

The question about bringing a certain term of the conditions to the counterparty’s attention was initially dealt with by English courts by looking at the conditions as a whole, and whether said conditions as a whole had been sufficiently drawn to a customer’s attention to make them part of the contract.^54^ Therefore, if the conditions were brought to the customer’s attention as a whole then the latter is seen as having assented to them even though he/she has not read them and does not have knowledge of their contents.^55^ In later stages, the courts started questioning whether a particular clause contained in these conditions should be treated exceptionally due to its unreasonableness.^56^ In an *obiter dictum* Lord Denning said that “Some clauses I have seen would need to be printed in red ink on the face of the document with a red hand pointing to it before the notice could be held to be sufficient”.^57^ In *Thornton v Shoe Lane Parking Ltd*,^58^ the Court held that where the enforcement of a particularly onerous condition is involved,^59^ the party seeking enforcement should show that it was fairly brought to the notice of the other party. Finally, in *Interfoto Picture Library Limited v Stiletto Visual Programmes Limited*,^60^ the Court found that a particular term contained in the conditions was ineffective because it was very onerous and the plaintiffs had failed to draw the defendant’s attention to said term.

The latter decision signifies a shift in that the promisor should make special efforts to make the consumer aware of the existence of terms which are difficult to understand, in order for them to be considered as being accepted (de Moor 1995, p. 263). In particular, Lord Bingham stated that the English common law has developed piecemeal solutions in response to demonstrated problems of unfairness, acting as functional equivalents to the good faith principle found in civil law systems. As Lord Justice Bingham put it in his interpretation of the good faith principle: “This does not simply mean that […contracting parties] should not deceive each other, a principle which any legal system must recognise; its effect is perhaps most aptly conveyed by such metaphorical colloquialisms as “playing fair”, “coming clean” or putting one’s cards face upwards on the table”.^61^

Protection from unfair terms, except from the technique of finding the unfair terms as having not been incorporated, was effectuated also through other methods such as the *contra proferentem* interpretation rule but also intervening on the ground of duress and undue influence (Howells 2017, p. 1910).^62^ Therefore, protection was mainly based on the promotion of procedural fairness (Howells 2017, p. 1910). Substantive control was introduced by the Unfair Contract Terms Act of 1977 but was focused mainly on exclusion and limitations of liability (Howells 2017, p. 1911). However, the introduction of Directive 93/13 by the EU was challenging for the UK, not only because all terms were subjected to the fairness test (with the exception of core terms if they are transparent), but because precisely this test was of a general nature, in contrast to the bright-line rules that the Unfair Contract Terms Act introduced (Howells 2017, p. 1912).

### The Challenges Posed by Directive 93/13 on the Common Law of Contract

Scholars suggest that whereas European law has affected the juristic outlooks of the English legal system (Palmer 2012, p. 71; MacMillan 2016, p. 426), at the same time, little of the substance of English contract law has changed directly as a result of membership in the EU (MacMillan 2016).^63^ Indeed, the most impactful EU legislative measure upon the common law of contract is the Directive on Unfair Terms in Consumer Contracts (Directive 93/13) which has posed the most challenges. Certain decisions of English courts have been criticized as being at odds with the European notion of the average consumer following the general assumption that the common law emphasizes *laissez-faire* 64 values which go against the EU’s general fairness test (assumed to have a protective ethic) (Howells 2017).^65^ Whereas an increasing number of decisions has recognized a duty of good faith as an implied term within the contract,^66^ such a shift cannot be considered as an embrace of the continental use of the concept, which is now less likely to take place given the UK exit from the EU (Pargendler 2018).^67^

Based on established caselaw from the CJEU it is for the national court to decide whether a contractual term satisfies the requirements of it to be regarded as unfair.^68^ Thus, the concept of fairness included in Article 3 of the directive is not subject to legal definition or interpretation at either the European or national level and it is a factual issue to be decided based on an assessment of the circumstances in each case. However, this will inevitably create a degree of uncertainty since the concept of fairness may be regarded as a discretionary concept that may yield different results in similar cases. Thus, the development of interpretive principles for such discretionary rule by the courts is necessary to develop a coherent framework (McKendrick 2019).^69^

Even though the directive is in its third decade of existence, it is still questionable whether the good faith requirement under Directive 93/13 is an independent test or whether it is linked to the significant imbalance test. Scholars also question whether the standard only requires clear conscience - which is similar to the interpretation that the Cypriot courts have given to the whole test found in Article 3 of the Directive - or whether the legitimate interests of the other party should be taken into account (Howells, Straetmans, 2017). Lower courts in Cyprus limit the test to a reference stating that in the absence of an allegation that the consumer entered into the contract involuntarily, the law can afford the consumer no defense to claims for unfairness, except for the terms in the grey list.^70^ However, the CJEU in *Aziz* stated about the good faith requirement that

[…]the national court must assess for those purposes whether the seller or supplier, dealing fairly and equitably with the consumer, could reasonably assume that the consumer would have agreed to such a term in individual contract negotiations.”^71^

On the other hand, the assessment of a significant imbalance requires an examination as to how a contract term influences the rights and obligations of the parties. As a result, there should be a comparison with the legal position of the consumer without the term and whether the term affects such default position.^72^ This means that the combination of the two tests indicate that there must be some substantive unfairness in the term under scrutiny (Howells, Straetmans, 2017). With regard to the annex that creates a grey list of terms, the CJEU has stated in *Nemzeti Fogyasztóvédelmi Hatóság v Invitel Távközlési Zrt*,^73^ that it is only an indicative and non-exhaustive list of terms which may be regarded as unfair, and the content of the annex may not suffice in itself to establish automatically the unfair nature of a contested term.^74^

The unfairness test, therefore, adopts the default rule comparison approach, which is closely aligned with the German law on content control of terms (Howells 2017, p. 1917). Furthermore, the rules of the directive reflect, to a great extent, the ‘informed consent’ model that civil law systems resort to when deciding whether a contract should be enforced (Beale 2012, p. 77; de Moor 1995).^75^ According to Anne de Moor, Directive 93/13 is drafted almost entirely from a civil law perspective, thus creating problems of integration within the common law conception of contract (de Moor 1995). This tension is even more apparent in the case of Cyprus as an analysis of the jurisprudence of the courts highlights (Mouttotos 2021), while it is evident that the approach of the courts is stricter than the one prevalent in English law.^76^ Unlike England and Wales, Cyprus did not introduce any safeguards with regard to standard form contracting, and a pure duty to read was imposed as a refusal of the legal system to intervene even in cases of undesirable behavior. English courts recognized the problems of insisting in the application of the will theory of contracts to standard form contracts, by emphasizing that freedom to contract must imply some room for bargaining (Tofaris, 2020).^77^ Cypriot courts’ approach towards questions of consent, in contrast, is based on the rule in *L’Estrange v Graucob*, where it was held that where a document containing contractual terms was signed, then, in the absence of fraud, misrepresentation and *non est factum*, the party cannot claim that he or she has not read the document and is, therefore, bound by such terms.^78^ Thus, the realization that there is a lack of bargaining and free will even in consumer cases where there is an asymmetry of bargaining power is not reflected in the Cypriot caselaw.

Cypriot courts are more limited also in terms of the range of materials upon which the courts could draw when seeking to interpret the contract.^79^ As the general approach of the common law, pre-contractual negotiations are excluded from admissible evidence as well as evidence of conduct subsequent to the making of the contract and statements of subjective intent (McKendrick et al. 2021).^80^ A testimony on subjective factors can only be accepted in an action for rectification.^81^ However, the interplay of Directive 93/13 with other consumer law directives that establish information obligations towards the supplier before the conclusion of the contract, may result in a shift in the rules regarding contractual interpretation, such as the relevance of pre-contractual negotiations. Moreover, the increased relevance of transparency when it comes to issues of assent to the terms of the contract through the extension of Article 5 of Directive 93/13,^82^ is challenging established principles of contract law in Cyprus such as the duty to read the terms of the contract (Benoliel, Becher, 2019).^83^

The recognition that there is a lack of adequate and effective means to prevent the continued use of unfair terms in consumer contract is also proven by the recent reasoned opinion sent to Cyprus by the European Commission.^84^ In particular, the Commission decided to send this opinion for the failure of Cyprus to properly implement and enforce EU law on unfair contract terms and unfair commercial practices. The Commission opened an infringement case in 2013 based on a series of complaints from EU citizens who bought real estate in Cyprus. Property developers, banks and lawyers had allegedly omitted to inform buyers about properties being free from encumbrances. The Commission found that the authorities in Cyprus were not effectively enforcing the two directives on consumer protection.^85^ This can be explained by the prevalence of the *caveat emptor* principle, and in general the rule that where the buyer had not been induced to sign a written contract by any misrepresentation, he/she was bound by the terms of the contract even if he/she has not read the document. Thus, the traditional concepts of signature and consent are prevalent in Cyprus law despite the extension of the transparency requirement provided under Article 5 of Directive 93/13 as well as other information duties imposed by European directives^86^ that challenge the traditional methods of incorporation of standard terms(especially) in consumer contracts.^87^

As a response to the failure to comply with the consumer protection directives, the legislature has passed a new statute for the consolidation, modernization and codification of consumer law in Cyprus, Law 112(I)/2021. Among its most important provisions is Article 63 establishing a private right of action, thus giving the consumer the right to file an action before a competent court for compensation and/or withdrawal from the contract and/or for reduction of the price. A rule of interpretation for all written terms is also adopted, providing that such written terms shall be formulated in a clear and intelligible manner and in case of doubt the meaning most favorable to the consumer shall prevail (*contra proferentem* rule).^88^ The Law gives more power to the sectorial regulator to investigate infringements of the law,^89^ something which may create fragmentation of the law since a different approach is often followed by the sectorial regulators when interpreting the same principles of contract law (Mouttotos 2021, pp. 250–257). However, the interpretation given by these authorities often reaches the courts through the injunctions’ procedure leading to a shift in the finding of unfair terms in consumer contracts by the courts, terms which have been earlier found to be enforceable.^90^ A limited number of decisions applies the directive in order to decide on the fairness of terms, whereas others tried to combine the question of unfairness with the vitiating factors of consent (mainly undue influence).

## Directive 93/13 Before Cypriot Courts – Interpreting the Directive in Line with the Common Law

The Unfair Contract Terms in Consumer Contracts Law (Law 93(I)/96) transposed the directive *verbatim*, with the exception of recent amendments not derived from the directive. The primary objective of the law is to protect the economic interests of consumers from unfair contract terms included in contracts with suppliers or service providers and consumers. This protection is to be safeguarded through statutory provisions and regulations.^91^ According to the District Court of Larnaca in *Director of Consumer Protection Service* (*Διευθυντή Υπηρεσίας Προστασίας Καταναλωτή*) *v Kimonos* (*Κίµωνος*),^92^ Directive 93/13 provides for individual and collective consumer protection from unfair terms. Article 7 of the Directive provides for the collective protection of consumers through the prevention of the continued use of unfair contract terms in non-individually negotiated contracts. Nonetheless, and despite the reference by the District Court of Larnaca to Article 7, the principle of privity of contract prevails, thus not allowing any generalized effect to individual decisions of unfairness (de Moor 1995).^93^

The interpretation of contractual terms by Cypriot courts is done with limited interference, allocating the risks based on the initial contractual stipulation following the four corners rule.^94^ In general, courts apply the parol evidence rule whereby as long as a writing is determined to be the final and complete agreement of the parties, extrinsic evidence are not allowed to supplement or contradict it, unless there is an ambiguity to be resolved (Bix 2012, p.60). Where determinations need to be made by the court in case of a dispute the four corners approach is one option for doing so by closely examining the document itself (Bix 2012). Courts in Cyprus have ruled that evidence produced to modify the content of the agreement that contradict the written text are not admissible.^95^

The limited interference of courts in the interpretation of written terms, even though such terms were written unilaterally, reflects the limited supervisory powers of the courts in the common law of contract in contrast to the civil law where a contract gives rise to obligation, the enforcement of which is out of the hands of the parties and into the supervisory powers of the courts (de Moor 1995). The *ex officio* control of unfairness that is provided under the protective regime of the Directive, is more closely oriented towards the civil law understanding regarding the supervisory powers of courts. In a great number of cases before Cypriot courts, consumers did not specifically point out the exact terms of the contract that they deemed unfair, despite claiming such unfairness. The courts, however, in such scenarios could request further clarifications by the litigating parties, in order to perform their role under the Directive which requires courts to raise out of their own motion issues of unfairness of terms.^96^

As mentioned above, Sect. 10 of the Contracts’ Law provides that all agreements are contracts if they are made by the free consent of the parties competent to contract for a lawful consideration and for a lawful object, but two or more persons are said to consent when they agree upon the same thing in the same sense (meeting of the wills).^97^ An underlying premise of Cypriot contract law is that a signed contract which was freely entered into, was enforceable including all of the terms within the document, irrespective of it being a standard contract used for a multitude of transactions.^98^ The onus is upon the complaining party to show a defect in the formation such as fraud, duress, misrepresentation and *non est factum*.^99^ Non-performance of a contract is governed by the doctrine of breach of contract, by providing damages, specific performance and injunctions, and the doctrine of impossibility of performance due to an unintentional weakness, where frustration is the only cause for justifying non-performance.^100^ Section 29 of Cap. 149 states the following: “Agreements the meaning of which is not certain, or capable of being made certain, are void”. According to the Court,^101^ this incorporates the common law rule that only agreements with terms which are certain are enforceable in law. Be that as it may, the use of defenses available in Cap. 149 in order to invalidate contractual terms on the grounds of unfairness has been very limited, perhaps even non-existent.

In particular, the undue influence defense was seen as potentially relevant to decide on the transaction’s unconscionability (Tofaris, 2020), however, the courts will require the existence of a relationship of trust to substantiate such a claim. In a case before the District Court of Larnaca,^102^ the Court reiterated the basic principles of contract law applicable in Cyprus, namely, that the consent of the parties is a precondition for the validity of a contract.^103^ A contract that is the product of violence or psychological coercion may be declared void.^104^ The common law of contract with the doctrine of duress and the law of equity with the doctrine of undue influence gives the right to a party to demand the cancellation of a contract lacking the component of consent.^105^ The law of equity cannot be used, however, to rescue a person from the effects of his/her imprudence, but only to prevent his/her victimization from third parties.^106^ Consent is considered free when it is not subject to coercion or duress, undue influence, fraud, misrepresentation and mistake.^107^ The burden of proof of recklessness or fraud is placed on the claimant who is required to bring forward sufficient and persuasive testimony to that end.^108^ In the case before the District Court of Larnaca, there was an agreement for the provision of investment services by way of securities with the debtors holding deposits with the financial institution for many years. This element established a fiduciary relationship and it was found that the financial institution abused the trust of the debtors in offering the financial product in question without considering the interests of its clients; failing also to provide adequate and satisfactory information (Mouttotos 2021, pp. 198–201). The Court found that due to the fiduciary relationship between the parties, a deviation from the rule of estoppel was justified, making reference to the decision in *Lloyds Bank Ltd v Bundy*.^109^ The claimants, according to the Court, placed their trust in the defendants as a leading financial institution and were justified in so doing, because of their own limited knowledge base for understanding complicated financial instruments. In that case, however, the Court found that due to the fiduciary relationship and the complexity of the investment product (contingent convertible bonds) the bank provided investment advice under Law 144(I)/2007 transposing the market in financial instruments Directive (MiFID)^110^ Therefore, there was a breach of a statutory duty.

A number of Cypriot decisions referred to the *dictum* by Lord Bingham in *Director General of Fair Trading v First National Bank plc*,^111^ in interpreting the good faith requirement. In that case Lord Bingham held that the requirement of good faith is one of fair and open dealing. Apart from reference to the cited *dictum*, however, Cypriot courts did not elaborate further to point out that this interpretation goes against the interpretation of good faith given by the Supreme Court of Cyprus as an absence of dishonesty.^112^ Regardless, as noted by the Fitness Check Study rapporteur for Cyprus, caselaw, which is rather limited, reveals the gaps in understanding the philosophy and aim of the law both on the part of courts and lawyers representing consumers.^113^ Caselaw of the CJEU found no representation in Cypriot caselaw until very recently. In fact, no court ruling existed where a thorough application of the principle-based approach of Article 3(1) of the Directive could be found. The test was limited to the search for bad faith or undue influence in agreeing on the terms.^114^ This signifies that the good faith requirement was construed in the English law sense of absence of dishonesty without taking into account the significant imbalance test. Therefore, under this interpretation, it seems that both procedural and substantive unfairness are not captured under the test.

Lower courts have repeatedly held that if the signature on the agreement is not disputed, the consumers are estopped from raising an issue of unfair contract terms; an interpretation which is more in line with the common law of contract.^115^ In *Maria Nicolaou* (*Μαρία Νικολάου*) *v Ellinas Finance LTD*,^116^ the appellants argued that certain - non-individually negotiated - terms in a credit contract were unfair since they caused a significant imbalance in the parties’ rights and obligations. The Court dismissed the arguments as it was not convinced that such an imbalance and non-negotiation existed.

At the same time, Cypriot courts tend to examine the unfairness of the contractual term without regard to the status of the contractual party as a consumer or business. For example, in *Euroinvestment & Finance v Schiza* (Σχίζα),^117^ the Court examined the unfairness of a term even though the contract involved an investment agreement. The defendant’s claim as to the unfairness of terms was dismissed by the court since throughout the duration of the agreement they had the power to terminate the agreement based on the law of contract. In reaching that conclusion, the Court examined the background of the defendants, noting that it involved an experienced investor that was aware of the risks involved in the agreement; an interpretation that can be contrasted to the interpretation given by the CJEU. In particular, the professional background of the party should not exclude a person from claiming unfairness, since the CJEU has held that a natural person practicing as a lawyer who concludes a credit agreement, which does not specify the purpose for which the credit is granted, may be regarded as a ‘consumer’.^118^ Nevertheless, in *I.S.G. Developers Limited, S. Yurmanov v Bank of Cyprus Public Company Ltd* (*Τράπεζας Κύπρου Δηµόσια Εταιρεία Λτδ*),^119^ the Cypriot court held that Law 93(I)/96 does not apply to the facts of the case since the claimants were not consumers, within the meaning of the Law, as they were a legal person rather than a natural person.

In cases before Cypriot courts, debtors have argued that the bank had an obligation to make sure that the borrowers received sufficient legal advice before signing the loan agreement or guarantee.^120^ The fact that such advice was not provided, according to the debtors, rendered the agreement voidable. Courts, in such cases, consider the background of the party arguing that such advice was essential before signing the agreement. In one such case, the fact that the debtor received copies of the agreement before signing and attempted to negotiate specific terms along with his profession as a certified accountant was crucial for the court to hold that the bank was not liable for not ensuring that the debtor received legal advice before signing the contract.^121^

Section 73(3) of the Contracts Law has been utilized as a defense to compensate for the loss that came as a result of the bank not selling the shares under an investment project financing contract.^122^ Section 73(3) stipulates that in estimating the loss or damage arising from a breach of contract, the means which existed of remedying the inconvenience caused by the non-performance of the contract must be considered. The court rejected the reasoning behind this argument since, in the facts of that case, the bank did not hold the shares in trust for the appellants, but they were rather given as collateral.^123^ In the same case, the appellants pleaded the defense of Sect. 67 that provides that: “if any promisee neglects or refuses to afford the promisor reasonable facilities for the performance of his promise, the promisor is excused by such neglect or refusal as to any non-performance caused thereby”. The court rejected the argument, as the appellants in the facts of the case were unwilling to perform their obligations under the contract, and it was not the bank that hindered such performance.

In the same vein, debtors use the defense of *non est factum* (not my deed). The defense of *non est factum* can be utilized by persons whose objective inability to understand the purpose of the particular document they are signing is due to blindness, illiteracy or some other disability. However, in *Saunders v Anglia Building Society*,^124^ it was maintained that in exceptional cases persons that are completely capable of understanding the nature of the document they are signing can plead a defense of *non est factum*.^125^

As a second precondition in order for a plea of *non est factum* to succeed, reasonable care must have been exercised as to the circumstances that have led the person pleading to sign the particular document.^126^ Finally, there must be a radical difference between that agreement which he signed and that which he thought he was signing.^127^ According to the judgment in *The Cyprus Development Bank LTD v Krini Evangelou Kyriacou*,^128^ in order for such a successful plea to be founded, undue influence must have been exercised by the counterparty or its agent. In *Bank of Cyprus v Theophanous*,^129^ the defendants raised the plea of *non est factum*; however, the court emphasized that in the facts of the case, the defendant knew that he was signing as a guarantor despite the fact that the document which he was signing was empty. Had he exercised reasonable care to ensure that the document was sufficiently completed before signing, he could have had a successful plea for *non est factum*. The *non est factum* defense is often accompanied by a claim of unfairness of contract terms under Directive 93/13. The courts examine the knowledge background of the person raising the defense in order to ascertain whether a contractual term is unfair.

In a recent judgment of first instance,^130^ the District Court of Nicosia found a number of clauses to be unfair and opaque and due to the plethora of such terms the contract was deemed invalid and not capable of continuing in existence without the unfair terms and thus unable to be performed. The Court referred to the caselaw of the CJEU^131^ and held that the signature of the consumer at the end of the contract is not evidence of negotiation of the contractual terms, thus going against previous caselaw where the courts rejected claims for unfairness as a result of the signature of the document.^132^ The Court indicated that the law sets a rebuttable presumption as to the existence of negotiation of terms with the burden being on the shoulders of the defendant/supplier. In its decision, the Court went on to examine each term separately, finding most of them as unfair and non-transparent. However, the actual test for examining the fairness of terms is not set out in clear terms in the judgment while the reasoning behind the finding of unfairness of non-transparent terms is nowhere to be seen. For example, the highest court in Greece, Areios Pagos, has reiterated in its caselaw that opaque or non-transparent terms conceal the real, legal and financial situation, hence creating the risk that the consumer will abstain from pursuing his or her rights or succumbing to rights that are falsely seen as belonging to the supplier.^133^

The District Court of Nicosia simply copies the text used by the Director for the Consumer Protection Service that states that the non-transparency of terms disturbs the contractual equilibrium in contravention of the principle of good faith. Be that as it may, this indicates the influence that sectorial regulators may have in the reception of continental interpretations of the good faith requirement of Directive 93/13. The Director for the Consumer Protection Service, in a number of decisions, has used jurisprudence from Greece and Germany in interpreting Directive 93/13.^134^ Greek courts, however, developed three principles for the assessment of the transparency of any contract term, thus including core terms. First, contract terms must be clear without any obscurity, and comprehensible. Comprehensibility is judged based on the subjective ability of the consumer to understand the term’s true meaning (Howells, Straetmans, 2017). The second principle is the determinable content of terms, which stipulates that there should be no vague terms (Howells, Straetmans, 2017). Lastly, the principle of foreseeability of terms prohibits surprise clauses/terms (Howells, Straetmans, 2017). The seller/supplier should ensure that these three principles are respected when drafting consumer contracts. Thus, reference to Greek caselaw should take into account the development of the principle of transparency/clarity in the Greek consumer protection regime (Karampatzos and Kotios 2019).

In holding the contract to be void, the District Court of Nicosia failed to explain the reasoning that led it to decide that the contract should be held void. The judge stated that in view of the plethora of the unfair and opaque terms the contract could not continue to be valid and enforceable. In the common law of contract, a document is usually be held void (as opposed to voidable) only when the element of consent to it is totally lacking.^135^ This means that the transaction which the document purports to effect is different in substance or in kind from the transaction originally intended.^136^ If such a legal analysis was done by the District Court, this would result in a further hybridization between EU law on consumer protection and contract law in Cyprus, through the use of the plea of *non est factum* and/or misrepresentation (Badenhoop 2020). That is to say that, due to the unfair terms, the document as signed was of a different character^137^ than what was actually intended to be signed by the consumer. In any case, nullity of contract (be it void, voidable or unenforceable contract) gives rise to the law of restitution and unjust enrichment.^138^ The CJEU held that since the nullity of the contract may have negative consequences for the consumer,^139^ national courts, in accordance with the principles of the law of contract, can substitute the deleted unfair terms with supplementary provisions of national law.^140^

## Conclusion

The principle-based approach found in Directive 93/13 as well as the caselaw of the CJEU reflect an understanding which is closely aligned to the continental handling of standard form contracting. Although it could undoubtedly be argued that the EU conception of fairness in contracts is highly instrumental (de Moor 1995) it is clear that following the financial crisis the CJEU has used the fairness requirement of the Directive to promote social goals such as consumer protection. In Cyprus there is an unwillingness to scrutinize the parties’ initial allocation, even though such allocation was done in a standardized manner by one party. Cyprus did not provide for consumer protection through statutory law or caselaw until the adoption of EU directives in the field. Consumers, therefore, had to resort to the general principles of private law in order to protect their interests (Hatzinestoros, Charalambous, 2016). However, Cap. 149 did not provide the necessary tools for dealing with substantive unfairness (Tofaris, 2020) and the Cypriot courts failed to draw inspiration from other common law jurisdictions as it pertains, in particular, to procedural fairness. In doing so, they refused to adopt the English courts’ analysis of the problems with standard form contracts and unfair terms, which can be attributed to the failure to enact legislative measures similar to the 1977 Act in the UK granting discretion to the courts to control contractual terms. Even in the absence of such a measure though, this is rather interesting since Cypriot courts showed readiness in interpreting and supplementing Cap. 149 with English law authorities. Be that as it may, even after the implementation of Directive 93/13, which filled a lacuna by providing for the judicial control of unfair terms in consumer contracts, courts still have trouble in performing their task in policing unfair terms.

Thus, the absence of similar statutory developments meant that judicial discretion in contractual matters was limited, with the courts having little interest in interfering in the terms of the contract. This has resulted in a discomfort by the courts about the power to interfere in such terms conferred on them under EU law. However, interpreting the terms of the contract via Directive 93/13 has become the cornerstone of consumer policy in the Union. With the increased focus on ensuring the effectiveness of Directive 93/13 as well as Directive 2005/29 on unfair commercial practices, it is expected that the approach of the courts will change, something that has gradually started to take place.

## Footnotes

1 Council Directive 93/13, 1993 O.J. (L 95) (EC) on Unfair Terms in Consumer Contracts.

2 See *Yam Seng Pte Ltd v International Trade Corp Ltd* [2013] EWHC 111 (QB); *Sjeikh Tahnoon v Ioannis Kent* [2018] EWHC 333, at 167 *et seq*.; and *D&G Cars Ltd v Essex Police Authority* [2015] EWHC 226 (QB) at 176.

3 See *Interfoto Picture Library Ltd v Stiletto Visual Programmes Ltd* [1987] EWCA Civ 6.

4 See also Giliker (2022) who refers to a shift in the caselaw in England and Wales towards the recognition of express and implied duties of good faith in relation to contractual performance.

5
*Royal Ris LTD v Municipality of Larnaca* (Δήµου Λάρνακας) [2005] 1 C.L.R. 1149.

6 In the case of Cyprus, the fairness test provided under the Directive was limited to the search for bad faith or undue influence in agreeing upon the terms of the contract. The judgment that is considered precedent as to the interpretation of the good faith requirement found in Law 93(I)/96 implementing the Unfair Contract Terms Directive is *Syrimi* (Συρίµη) *v Pancyprian* (Οργανισµός Χρηµατοδοτήσεως Παγκυπριακή) [2010] 1 C.L.R. 1131. The Supreme Court interpreted the good faith requirement as an absence of bad faith or dishonesty which is different than the approach taken in the UK, where courts have not sought to interpret the requirement from a national perspective (See McKendrick 2008, p. 501; Howells 2017, pp.1922–1926).

7 [1934] 2 K.B. 394. Caselaw from Cypriot courts makes reference to *L’Estrange v Graucob* as setting a principle of law that dictates that a party, when signing an agreement, is bound by it, even if said party has not read it, or even if it is unaware of its effect on the law. See *Kkazanou* (Κκαζανου) *v Cyprus Development Bank* (Κυπριακής Τράπεζας Αναπτύξεως) ECLI:CY:AD:2020:A167; *Cyprus Development Bank v Evangelou Kyriakou* [1989] 1 C.L.R. 96; *Toujikian v Cyprus Popular Bank* (Λαϊκής Κυπριακής Τράπεζας) [2003] 1 C.L.R. 1240; *Zachariade* (Ζαχαριάδη) *v Universal Life Insurance* ECLI:CY:AD:2019:A145: in this judgment the Supreme Court explicitly said that it is a basic rule of the common law of contract that a party signing an agreement is bound by it.

8 See below, subsection i.

9 For the impact of the pandemic on the economy at large see Price Waterhouse Coopers Cyprus “COVID-19: Impact of the pandemic on the Cyprus Economy” 13 May 2020.

10 The term is used to describe the 1974 invasion and eventual occupation and the *de facto* division of the island that began from 1963.

11 See Courts of Justice Law, L. 14/60, 1960, Art. 29.

12 See *supra* note 2.

13 See Section III of the article.

14 See to that effect note 48.

15 Article 3(1) Directive 93/13.

16 See for example, the Uniform Commercial Code in the United States of America, § 1-304.

17 Sect. 18, Chap. 149, (Cap. 149) Contracts’ Law, L. 24/30.

18
*Yam Seng Pte Ltd supra* note 3.

19 See page 23 of the article.

20 This is seen, for example, from the transplantation of the Sale of Goods Act of 1979. New legislation on Sale of Goods enacted in 1994 (L. 10(I)/1994) replaced the English Sale of Goods Act of 1893 (applicable under Sale of Goods Law (Cap. 267), L.25/1953) with the transplantation of the English Sale of Goods Act of 1979.

21 Despite the fact that the CISG entered into force over a decade ago in Cyprus – which could have led to an autonomous source of law with its own interpretation and application, separate from the common law – the CISG has yet to generate any caselaw. It may be argued that this reflects a preference towards the English law of sale, which is preferred as the governing law in international sales (Yan 2011; Mouttotos 2019).

22 United Nations Convention on Contracts for the International Sale of Goods, 1980. According to Rogowska, the English legal system has achieved wide international acceptance and familiarity since, despite the advantages of the CISG, business parties worldwide have frequently incorporated English law into their international sales contracts (Rogowska 2013).

23 Despite the fact that the CISG entered into force over a decade ago in Cyprus – which could have led to an autonomous source of law with its own interpretation and application, separate from the common law – the CISG has yet to generate any caselaw. It may be argued that this reflects a preference towards the English law of sale, which is preferred as the governing law in international sales (Yan 2011; Mouttotos 2019).

24 Chapter 149, (Cap. 149) Contracts’ Law, L. 24/30.

25 Sect. 2 (1) Cap. 149.

26 Sect. 10 of Cap. 149.

27 Sect. 13 of Cap. 149.

28 Courts in Cyprus have referred to the dictum of Lord Justice Clarke in *RTS Flexible Systems Ltd v Molkerei Alois Muller GmbH and Co KG* [2010] U.K.S.C. 14: “The general principles are not in doubt. Whether there is a binding contract between the parties and, if so, upon what terms depends upon what they have agreed. It depends not upon their subjective state of mind, but upon a consideration of what was communicated between them by words or conduct, and whether that leads objectively to a conclusion that they intended to create legal relations and had agreed upon all the terms which they regarded or the law requires as essential for the formation of legally binding relations”.

29 The validity of mutual declarations under continental law is based upon good faith and the principle of *pacta sunt servanda* (see Chloros 1968, p. 139).

30 See Sect. 14 *et seq.* of Cap. 149.

31 Sects. 15–18 of Cap. 149.

32 Tofaris points out that Indian courts have made use of Sect. 23 - which provides for the public policy exception – to strike down contractual terms as contrary to public policy. In particular Sect. 23 of Cap. 149 (which is identical in both texts) provides that: “The consideration or object of an agreement is lawful, unless (a) it is forbidden by law; or (b) is of such a nature that, if permitted, it would defeat the provisions of any law; or (c) is fraudulent; or (d) involves or implies injury to the person or property of another; or (e) the Court regards it as immoral, or opposed to public policy”. See Tofaris, 2020, p.115 *et seq*.

33 Chapter 50.

34 Preamble to the Act.

35 See *Constantinou v Panayides* [1984] 1 C.L.R. 466: where it was clarified that the common law finds application in Cyprus in light of the provisions of the Courts of Justice Law. The Court explained that what is applicable is the common law not as it may have been modified by English statute: “What is applicable in Cyprus is the common law, not the common law as it came to be modified by statute in England. Section 29(1)(c) incorporates the common law (see Law 14/60), not English Law as applicable in England at any one time. The enticement was a cause of action recognised by the common law, in fact it is a cause that was recognised as actionable many centuries ago.”

36 Legislation enacted during the colonial era is referred to by Chapter (cap.) number while legislation post-independence is referred to by their short title and year of publication. Legislation enacted during the colonial era constituted an effective codification of common law principles in their respective fields and most of the legislation is still in force today.

37 As it is pointed out, it is not unlikely for lawyers to “cheat” by resorting to authorities and subsequent caselaw, thus leading the Cyprus courts to accept English law – as modified by statute – to constitute the common law (Hatzimihail, 2015).

38 Sect. 2(1) Cap. 149.

39
*Myrianthousis v Petrou* [1956] (V2) 1 C.L.R. 32: on past consideration; *Maison Jenny Ltd v Krashias Footwear Industry Ltd* [2002] 1 C.L.R. 1156: on frustration [in Greek].

40 English caselaw dealing with Directive 93/13 reaches the courts mainly through the efforts of the Director of the Consumer Protection Service, making reference to the case of *Director General of Fair Trading v First National Bank plc* [2002] 1 A.C. 481 and in particular Lord Bingham’s dictum at para. 17: “The requirement of good faith […] is one of fair and open dealing. Openness requires that the terms should be expressed fully, clearly and legibly, containing no concealed pitfalls or traps. Appropriate prominence should be given to terms which might operate disadvantageously to the consumer. Fair dealing requires that a supplier should not, whether deliberately or unconsciously, take advantage of the consumer’s necessity, indigence, lack of experience, unfamiliarity with the subject matter of the contract, weak bargaining position […]”. This dictum was found in a first-instance decision before the District Court of Larnaca. See *Director of Consumer Protection Service* (Διευθυντή Υπηρεσίας Προστασίας Καταναλωτή) *v Kimonos* (Κίµωνος) ECLI:CY:EDLAR:2018:A129. The Court, in interpreting the good faith requirement of the directive, used the *dictum* by Lord Bingham but did not go further than that. The Court found that the terms obliging the buyer of land to repay the total amount of the sale price upon completion of the works on the property; to have insured the property until a title deed is issued in his name; to pay 9% interest in case of delay in the payment of installments of the sale price were unfair since they exclude or restrict the consumer’s statutory rights vis-à-vis the seller or the supplier; they impose disproportionately high compensation on the consumer who fails to fulfill his/her obligations; and they oblige the consumer to fulfill all his/her obligations, while the seller or supplier does not fulfill his/her own.

41 [1987] 1 C.L.R. 583.

42 [1987] 1 C.L.R. 297.

43 [1982] 1 C.L.R. 499.

44 [1843-60] All E.R. Rep. 461. The judgment sets the rule that “[w]here two parties have made a contract, which one of them has broken, the damages which the other party ought to receive in respect of such breach of contract should be such as may fairly and reasonably be considered either arising naturally, i.e. according to the usual course of things, from such breach of contract itself, or such as may reasonably be supposed to have been in the contemplation of both parties at the time they made the contract, as the probable result of the breach of it”.

45 See *Heron II* [1967] 3 All E.R. 686; *Soleada S.A. v Hamoor Tanker Corporation Inc.* [1981] 1 All E.R. 856.

46 According to Article 5 of Directive 93/13 “terms must always be drafted in plain, intelligible language. Where there is doubt about the meaning of a term, the interpretation most favourable to the consumer shall prevail”. Article 4(2) provides that “[a]sessment of the unfair nature of the terms shall relate neither to the definition of the main subject matter of the contract nor to the adequacy of the price and remuneration […] in so far as these terms are in plain intelligible language”. Thus, terms which are not assessable for their fairness are terms that describe the main subject matter of the contract or the quality/price ratio of the goods or services supplied as long as they are transparent (Recital 19 in the preamble to the directive).

47 C-34/18 *Ottília Lovasné Tóth v ERSTE Bank Hungary Zrt* [2019] ECLI:EU:C:2019:245 Opinion of Advocate General Hogan.

48 C-191/15 *Verein für Konsumenteninformation v Amazon EU Sàrl* [2016] ECLI:EU:C:2016:612.

49 See for example, *Cooperative Bank (Συνεργατική Εταιρεία Διαχείρισης Περιουσιακών Στοιχείων) v Neokleous (Νεοκλέους)* ECLI:CY:EDLEM:2021:A65.

50 SCHEDULE 2, Unfair Contract Terms Act 1977, c. 50, “Guidelines” for Application of Reasonableness Test.

51 [1973] 1 All E.R. 193, by Lord Denning M.R. (at p. 200).

52
*Kokkalos v Karayiannis* [1975] 1 C.L.R. 377.

53 See *STEFANOS & ANDREAS COLD STORES TRADING LIMITED v Soft Drinks Company Kean (ΕΤΑΙΡΕΙΑΣ ΑΝΑΨΥΚΤΙΚΩΝ ΚΕΑΝ ΛΙΜΙΤΕΔ)* [1998] 1 C.L.R. 2335.

54 See *Parker v South Eastern Railway Company* [1877] 2 CPD 416.

55
*Ibid*.

56 See *J. Spurling Limited v Bradshaw* [1956] WLR 461.

57
*Ibid*, at 466.

58 [1971] 2 QB 163.

59 In that case the condition was restrictive of statutory rights.

60
*Supra* note 5.

61
*Ibid*, at 439.

62 On the latter issue see *Barclays Bank plc v O’Brien* [1994] 1 AC 180. The bank had knowledge that the defendants signed the documents without any warning of the risks or any recommendation to take legal advice.

63 Sir Geoffrey Vos argues that European law does not have an impact on the common law since the former is mostly about mutuality between Member States and the regulation of sectors affecting the single market and trade between the Member States. According to this view, European law has nothing specifically to do with the private law that Member States use to resolve disputes between individuals or businesses (Vos 2019).

64 However, see Richard Epstein’s essay challenging the affinity of *laissez-faire* philosophy with the rigid and mechanical nature of contract law, rather being more connected to the idea of ‘security of exchange’ (Epstein 1997).

65 See *Office of Fair Trading v Abbey National plc* [2010] 1 A.C. 696.

66 See *Yam Seng Pte Ltd supra* note 3.

67 On 31st January 2020 the United Kingdom has formally left the European Union, thus becoming a third country to the EU. February 1st 2020 marks the beginning of a transitional period which lasted until the 31st of December 2020 providing more time for businesses and citizens to adapt. For more details see https://www.consilium.europa.eu/en/press/press-releases/2020/01/30/brexit-council-adopts-decision-to-conclude-the-withdrawal-agreement/.

68 C-237/02 *Freiburger Kommunalbauten GmbH Baugesellschaft & Co. KG v Ludger Hofstetter* ECLI:EU:C:2004:209.

69 See also Ewan McKendrick. Doctrine and Discretion in the Law of Contract Revisited. 2019. *The Chinese Journal of Comparative Law* 7: 4 referring to statutes containing discretionary rules.

70 For example, *National Bank of Greece (Cyprus) (Εθνική Τράπεζα της Ελλάδος (Κύπρου) Λτδ) v Theokli & Levadioti Real Estate Companies (Θεοκλή & Λειβαδιώτη Κτηµατικές Επιχειρήσεις Λτδ)* ECLI:CY:EDLAR:2014:A41.

71 C-415/11 *Aziz* ECLI:EU:C:2013:164 para. 69.

72
*Ibid*, para. 68.

73 Case C-472/10 *Nemzeti Fogyasztóvédelmi Hatóság v Invitel Távközlési Zrt* ECLI:EU:C:2012:242.

74
*Ibid*, paras. 25–26.

75 Anne de Moor argues that a contract is an accord of wills in the civil law, hence, requiring that the parties were in mutual agreement and not just entered into the contract voluntarily or intentionally. However, she argues that “[i]t would be contrary to the objective principle in English contract law […] for the courts to ensure that the substantive entitlements and liabilities so created are necessarily those on which the parties were in agreement”. Thus, the provision by the Directive of non-negotiated contractual terms mirrors the civilian notion of accord of wills (de Moor 1995).

76 In the case of Cyprus, the fairness test provided under the Directive was limited to the search for bad faith or undue influence in agreeing upon the terms of the contract.

77 See for example *Suisse Atlantique Société d’ Armament SA v NV Rotterdamsche Kolen Centrale* [1967] 1 AC 361, Lord Reid.

78 See the English case that established the rule, *supra* note 8.

79 See, for example, *Chrysostomou (Χρυσοστόµου) v Hellenic Bank Public Company Ltd (Ελληνική Τράπεζα Δηµόσια Εταιρεία Λτδ)* ECLI:CY:EDLEF:2016:A532: in that case the Court pointed out that the source for the quest and basis upon which the various terms of the agreement will be judged as to their fairness is the written text itself. Based on their interpretation it will be decided whether said terms comply with the transparency rule and meet the conditions for consumer protection.

80 For the rules of contractual interpretation as set by the Supreme Court in Cyprus see *Theologou (Θεολόγου) v Real-Estate Company (Κτηµατικής Εταιρείας)* [1998] 1 C.L.R. 407. The Court stated that the text of a contractual provision read in the context of the agreement constitutes the fundamental rule of contractual interpretation. The text is interpreted using the criterion of the average reasonable person. The background of the agreement may be used in order to interpret the meaning of the text excluding the negotiations as well as subjective, unilateral intentions of the parties.

81
*STEFANOS & ANDREAS COLD STORES TRADING LIMITED v Soft Drinks Company Kean (ΕΤΑΙΡΕΙΑΣ ΑΝΑΨΥΚΤΙΚΩΝ ΚΕΑΝ ΛΙΜΙΤΕΔ)* [1998] 1 C.L.R. 2335.

82 See for example Article 6a(1) of the Consumer Rights Directive 2011/83 (adopted by the Modernization Directive, A New Deal For Consumers COM (2018) 183 final), which creates duties for the provision of information to consumers.

83 Deriving from the *caveat emptor* principle. On the duty to read as an important building block of the common law and the challenges posed by standard form contracts (Benoliel, Becher, 2019).

84 See February 2021 Infringements Decisions at https://ec.europa.eu/commission/presscorner/detail/en/inf_21_441.

85 European Commission, Infringements decisions, July infringements package: key decisions, 25 July 2019.

86 See Directive 2014/17 EU on credit agreements for consumers relating to residential immovable property.

87 But see also the ‘red hand’ rule under the common law that provides that when a term is particularly onerous to the consumer and the latter did not know of the term in question, the trader should do what is reasonably sufficient to bring to the consumer’s notice the term in question. *Thornton v Shoe Lane Parking Ltd* [1971] 2 Q.B. 163.

88 Article 52, Law 112(I)/2021.

89 See Article 57, Law 112(I)/2021.

90 For example, the contractual clause indicating for purposes of calculating the interest rate provided for a 360-day year and was held unfair by the Ombudsman, arguably applying “the principles of Cap. 149”. However, the courts in similar cases, which claimed the unfairness of a clause providing for the use of a 360-day year, held otherwise. See *Archbold Investments Ltd v Λαϊκής Κυπριακής Τράπεζας Λτδ* [2006] C.L.R. 108. In a later judgment by the Supreme Court, however, the 360-day divisor was found as unfair since it burdened the consumer with an extra 1.3889% interest each day. See *Βογαζιανος v Τράπεζας Κύπρου* [2011] Civil Appeal 281/2006.

91 Preamble to Law 93(I)/96.

92
*Supra* note 39.

93
*Tsitsos (Τσιτσος) v Emporiki Bank Cyprus* ECLI:CY:EDLAR:2021:A39: where the Court rejected the claim of similar terms being held unfair by the Consumer Protection Service.

94 See *Tsialis* (Τσιαλής) *v Hatziandreou* (Χατζηανδρέου) [2000] C.L.R. 1250. This case involved the implication of a term that was not written within the document. The Court made reference to *Gwyin v Neath Canal Co* [1865] L.R. 3 Ex. 209 where in page 215 it was said that: “The result of all the authorities is, that when a court of law can clearly collect from the language within the four corners of a deed, or instrument in writing, the real intentions of the parties, they are bound to give effect to it by supplying anything necessarily to be inferred from the terms used, and by rejecting as superfluous whatever is repugnant to the intention so discerned”.

95 See *Efthimiou* (Ευθυµίου) *v Demetriou* (Δηµητρίου) [2001] C.L.R. 1721; *Saab & Another supra* note 42.

96 See for example *N.A. Nikolaou (Helios Estates) Ltd v Bank of Cyprus* (Τράπεζα Κύπρου) ECLI:CY:EDPAF:2018:A108. See also *Ellinas Finance Public Company Ltd v Hatzipanagiotou* (Χατζηπαναγιώτου) ECLI:CY:EDLEF:2020:A382. In the latter case, the Court went on to examine the unfairness despite the failure to mention it in the application. However, the Court stated that terms cannot be held unfair if there is no testimony that rebuts the presumption of existence of good faith by the supplier. This is a false understanding of the test contained in Article 3 of the Directive, since it good faith is interpreted as an absence of dishonesty.

97 Sects. 10 and 13 of Cap. 149. Section 13 gives the definition of consent.

98 See, for example, *Koullapis* (Κουλλάπης) *v National Bank of Greece (Cyprus)* (Εθνική Τράπεζα της Ελλάδος (Κύπρου)) ECLI:CY:AD:2015:A751: where the Supreme Court in being called to examine the fairness of the terms of the whole credit facility agreement noted that the appellant (debtor) was not forced into signing the standardized contract and enter into the agreement, while the bank did not exercise bad faith or undue influence upon the debtor.

99 See Sect. 19, Cap. 149. See also Order 19 Rule 5 of the Civil Procedure Rules where it is provided that: “In all cases in which the party pleading relies on any misrepresentation, fraud, breach of trust, willful default, or undue influence, full particulars thereof shall be stated in the pleading. In the case of fraud, the alleged fraudulent acts must be specially set out and it must be averred that such acts were done fraudulently”. See also *Xenophontos* (Ξενοφώντος) *v Bank of Cyprus* (Τράπεζας Κύπρου) ECLI:CY:EDLEF:2018:A422.

100 See Sect. 51 *et seq.* of Cap. 149 and in particular Sect. 56 which establishes the doctrine of impossibility. In particular, it is provided that “[a] contract to do an act which, after the contract is made becomes impossible, or, by reason of some event which the promisor could not prevent, unlawful, becomes void when the act becomes impossible or unlawful” (paragraph 2 of Sect. 56).

101
*Sekavin* [1987] *supra* note 41.

102
*Georgiou* (Γεωργίου) *v Bank of Cyprus* (Τράπεζα Κύπρου) [2018] ECLI:CY:EDLAR:2018:A138.

103 Sects. 13–14, Cap. 149.

104 Sect. 15, Cap. 149.

105 Duress, including economic duress, has been recognized by caselaw in Cyprus; see *Koutas* (Κούτας) *v Municipality of Nicosia* (Δήµου Λευκωσίας) 1 C.L.R. 516.

106
*Socratis Savva* (Σωκράτης Σάββα) *v Socratous Panikou Sivitanidi* (Σωκράτους Πανίκου Σιβιτανίδη) [1998] 1 C.L.R. 1602.

107 Sect. 14 of Cap. 149.

108 The District Court of Larnaca reiterated that in order for the claimant to have the right to cancel the contract and/or be subject to compensation should prove that: (i) the representation was material in that it would have influenced the decision of a reasonable person in concluding the contract; (ii) the consent of that party was given based on the misrepresentation; and (iii) the party influenced by the misrepresentation could not have discovered the truth through the exercise of reasonable care and diligence.

109 [1975] Q.B. 326.

110 Directive 2004/39/EC superseded by Directive 2014/65/EU. The Cypriot Law was itself superseded by Law 87(I)/2017.

111 [2001] UKHL 52; [2002] 1 AC 481.

112 See caselaw referred to in note 39. For the decision of the Supreme Court that set the precedent see note 113.

113 Study for the Fitness Check of EU consumer and marketing law, Final report Part 3 – Country reporting, Prepared by Civic Consulting. May 2017. 207.

114
*Syrimi* (Συρίµη) *supra* note 7 is the precedent followed when applying the test provided under Article 4(1) of the Directive.

115 Study for the Fitness Check of EU consumer and marketing law, Final report Part 3 – Country reporting, Prepared by Civic Consulting. May 2017. 208. See *Charalambous* (Χαραλάµπους) *v Achilleos et al.* (Αχιλλέως κ.α.) [2001] 1 (Β) C.L.R. 1058, 1068; *Theologos et al.* (Θεολόγος κ.α.) *v Nemesis Real Estate Company* (Κτηµατική Εταιρεία Νέµεσις Λτδ) [1998] 1 C.L.R. 407; *Poliphimos Hotels LTD* (Πολύφηµος Χοτέλς Λτδ) *v Moutsi* (Μούτση) [2000] 1 C.L.R. 1809. See also *Iakovides* (Ιακωβίδης) *v Hellenic Bank* (Ελληνική Τράπεζα) ECLI:CY:EDLAR:2019:A130. See also the case of *Georghiou* (Γεωργίου) *v Bank of Cyprus* (Τράπεζα Κύπρου) ECLI:CY:EDLAR:2018:A138. where the bank argued that the debtors were estopped from arguing that the contract is void since they signed the document themselves. See also *Bank of Cyprus* (Τραπεζα Κύπρου) *v Varbedian*, ECLI:CY:EDLAR:2019:A167 where the claim by the defendants that they could not read Greek, so they did not understand what they were signing was rejected as a result of the failure to be added in the case file. The Court, however, said that even if it was covered the burden is upon the person raising the claim to overturn the conclusion of the signature, which indicates that they at least know how to read.

116 [2013] 1 C.L.R. 2392.

117 ECLI:CY:EDLEF:2014:A184.

118 C-110/14 *Horațiu Ovidiu Costea v SC Volksbank România SA* [2015] ECLI:EU:C:2015:538.

119 ECLI:CY:EDLEF:2016:A383.

120
*National Bank of Greece supra* note 69. Reference was made to *Royal Bank of Scotland v Etridge* (No 2) [2002] 2 AC 333.

121
*Ibid*.

122 See *Ellinas Finance LTD v Sofia Matsi* (Σοφίας Μάτση) [2009] App. No. 8634/02 and the appeal before the Supreme Court, *Matsi* (Μάτση) *v Ellinas Finance* [2012] 1 C.L.R. 2400.

123
*Gregoriou* (Γρηγορίου) *v Euroinvestment & Finance LTD* [2011] 1 C.L.R. 2229.

124 [1971] A.C. 1004.

125 According to Lord Reid (ibid., at 964): “The plea of *non est factum* obviously applies when the person sought to be held liable did not in fact sign the document. But at least since the sixteenth century it has also been held to apply in certain cases so as to enable a person who in fact signed a document to say that it is not his deed. Obviously, any such extension must be kept within narrow limits if it is not to shake the confidence of those who habitually and rightly rely on signatures when there is no obvious reason to doubt their validity. Originally this extension appears to have been made in favour of those who were unable to read owing to blindness or illiteracy and who therefore had to trust someone to tell them what they were signing. I think it must also apply in favour of those who are permanently or temporarily unable through no fault of their own to have without explanation any real understanding of the purport of a particular document, whether that be from defective education, illness or innate incapacity. But that does not excuse them from taking such precautions as they reasonably can. The matter generally arises where an innocent third party has relied on a signed document in ignorance of the circumstances in which it was signed, and where he will suffer loss if the maker of the document is allowed to have it declared a nullity. So, there must be a heavy burden of proof on the person who seeks to invoke this remedy. […] I do not say that the remedy can never be available to a man of full capacity. But that could only be in very exceptional circumstances: certainly not where his reason for not scrutinizing the document before signing it was that he was too busy or too lazy. In general, I do not think he can be heard to say that he signed in reliance on someone he trusted. But, particularly when he was led to believe that the document which he signed was not one which affected his legal rights, there may be cases where this plea can properly be applied in favour of a man of full capacity” [emphasis added].

126 See ibid.

127 Lord Reid at 964, *Saunders supra* note124.

128 [1989] 1 C.L.R. 96.

129
*Τράπεζα Κύπρου Δηµόσια Εταιρεία ΛΤΔ v Θεοφάνους* [2014] ECLI:CY:EDPAF:2014:A80.

130
*Director of Consumer Protection Service* (Διευθυντή Υπηρεσίας Προστασίας του Καταναλωτή) *v Société General Bank Cyprus Limited* ECLI:CY:EDLEF:2021:A110.

131 C-452/18 *XZ v Ibercaja Banco, SA* ECLI:EU:C:2020:536.

132 The decision goes against previous caselaw from the Supreme Court regarding the day-count convention set at 360 days instead of 365. The 360-day divisor was found as unfair, whereas the Supreme Court has accepted such a term in the case of *Archbold Investments Ltd v Λαϊκή Κυπριακή Τράπεζα* [2006] 1 C.L.R. 1084.

133 Areios Pagos, decision no 430/2005 ΕλλΔνη 2005, 802 *et seq*.

134 See Decision of the Director of the Consumer Protection Service 2018/01, 8.13.10.26.4.4.1.

135 See to that effect Lord Justice Wilberforce in *Saunders supra* note 123.

136
*Ibid.*

137 “Character” of the document is used in *Saunders supra* note 123 interchangeably with “actual contents” of a document.

138 See also Sects. 62–67 of Cap. 149.

139 See for example Joined Cases C482/13, C484/13, C485/13 and C487/13, *Unicaja Banco, SA* ECLI:EU:C:2015:21 para. 33.

140 See C-26/13. *Kásler and Káslerné Rábai* ECLI:EU:C:2014:282 paras. 80–81.
